# The Hospital Isolation of Infectious Diseases

**Published:** 1913-09-27

**Authors:** H. Franklin Parsons

**Affiliations:** late Assistant M. O., Local Government Board.


### THE HOSPITAL ISOLATION OF INFECTIOUS DISEASES.*

### IV.—Administrative Difficulties in Small Isolation Hospitals.

By H. FRANKLIN PARSONS, M.D., D.P.H., late Assistant M. O., Local Government Board.

In the previous article (The Hospital, August 9, 1913), in referring to the importance of bed-isolation and aseptic nursing for the prevention of cross-infections—including the interchange of secondary infections among patients admitted for the same primary disease—allusion was made to the difficulty of securing a staff of nurses competent to carry out these methods in small hospitals, which might be often void of patients, and to the consequent advantage, in the case of small districts, of combination for the provision of a joint hospital of convenient size over the establishment of a small separate hospital for each district. There are, indeed, many other advantages in combination. To quote from a Memorandum issued by the Local Government Board: "The unnecessary multiplication of small hospitals is to be avoided on grounds both of economy and efficiency. As compared with that of several smaller hospitals, the establishment of a single hospital containing an equal number of beds saves the cost of duplicating various buildings, appliances, and officers; it facilitates the classification of patients according to the diseases from which they are suffering; and it enables a more efficient staff to be maintained, since the hospital is less likely to remain empty for considerable periods." To this it may be added that there is only one site to be obtained instead of several, with a larger area for selection.

The chief objection usually raised to the establishment of joint hospitals for combinations of districts is that it involves the conveyance of patients over longer distances. But with the present improved state of the roads in most parts of the country, with modern improvements in means of communication and transport—*e.g.* telephones, bicycles, motor cars and ambulances, and wheels with indiarubber and pneumatic tyres—together with increased appreciation on the part of the public of the advantages of hospital treatment, it is found that the area which a hospital may conveniently serve is much larger than was formerly supposed. It is the moving of a patient dangerously ill which constitutes a risk, rather than the length of journey in a smoothly-running ambulance over good roads; and in practice distances of ten to twelve miles have not prevented the use of a hospital for cases of scarlet fever; while smallpox patients have been removed without harm by road for distances of twenty, or even thirty, miles. Nor is there any reason for supposing that, assuming due care, greater danger to the public would arise from the conveyance of patients to the hospital from longer distances, or from the larger number under treatment in one place; on the contrary, the aggregate danger from a number of small hospitals with makeshift arrangements might be expected to be greater than that from a single well-equipped and well-managed one.

It may perhaps be thought that increasing the area from which patients are drawn might increase the risk of cross-infection, inasmuch as different infections, or different strains of infection, would be more likely to be present in cases derived from a larger area than in those from one locality only. But it cannot be safely assumed that in a locality, however small, only one infection will be present at a time. Even in villages mixed outbreaks of scarlet fever and diphtheria, and outbreaks of septic scarlet fever may occur; and it has often happened, in cases where a hospital served only a single small urban or rural district, that a patient with diphtheria or enteric fever, whose removal to hospital was urgently needed, could not be taken in because the hospital was "full of scarlatina cases." In a hospital for a combined area the larger number of wards would afford more facility for isolating cases of different kinds.

The present article does not, of course, apply to London, which is served by the hospitals of the Metropolitan Asylums Board, nor to the large towns, most of which have provided efficient hospitals.

The power given to local authorities by Section 131, Public Health Act, 1875, to provide "hospitals or temporary places for the reception of the sick" carries with it the power—and, indeed, the moral duty—to provide for the sick admitted into such places food, nursing, medical attendance, and other requisites which their condition may require; and probably a legal liability would rest on the local authority in the event of harm resulting from their omission to supply any such requisite. But the law lays down no specific requirements in the matter, and the Local Government Board have, outside London, no control over the administration of isolation hospitals, such as they have over Poor-Law institutions, including the hospitals of the Metropolitan Asylums Board. The County Council have some indirect control, inasmuch as they are empowered by the Isolation Hospitals Acts to contribute to the expenses, including establishment expenses, of an isolation hospital where they deem it expedient so to do for the benefit of the county, and they may make it a condition of their contribution that the hospital is properly equipped, and provided with a sufficient and suitable staff.

In cases in which the sanction of the Local Government Board is required for the erection of an isolation hospital—as, for instance, when it is to be erected by means of a loan—it is the practice of the Board to require, as a condition of their sanction, that quarters for staff, laundry, and other administrative accommodation shall be provided on a scale reasonably sufficient for the needs of the hospital at times when the ward accommodation provided is in full use. But the Board have no power to prescribe the number or qualifications of the nurses or other officers, or to intervene in the internal administration of an isolation hospital, and it is only in rare cases of scandal or complaint that they

* Previous articles appeared on June 21, July 19, and August 9.

have directed inquiry into the management of one. Moreover, it is only in certain cases—*e.g.* where a loan is sought—that approval by the Local Government Board of the structure of an isolation hospital is necessary. The local authority, if they pay for it out of current revenue, may provide hospital accommodation of whatever kind they please without having to obtain the Board's consent, and even without the Board's knowledge, except for such information as it may be able to obtain incidentally from the reports of the medical officer of health, or from those of its own inspectors who may have visited the district.

Temporary or extemporised hospitals are, as was long ago pointed out by Sir R. Thorne, almost invariably unsatisfactory; they are often not ready for occupation until the immediate cause of their erection has passed by; the accommodation provided is often of a very indifferent sort, and especially is usually deficient as regards sleeping rooms for nurses and other administrative accommodation, and they commonly fail to meet the permanent requirements of the district. The ready-made hospitals are commonly deficient in space per bed and in administrative accommodation, and are very inflammable and liable to be wrecked by storms. Existing buildings designed for other purposes are rarely found well adapted, even by the aid of considerable alterations, for the reception of patients, especially of those suffering from different infectious diseases. With regard to tents or huts erected as occasion may require, before they are ready to receive patients a site has to be found on which permission to erect them can be obtained, which is often not easy; the site has to be prepared, a water supply and means of drainage provided, furniture and other necessary equipment procured, nurses engaged, and supplies arranged for, and all these things take time and cost money. It is found with all such temporary arrangements that the local authority are often unwilling to incur the expense of bringing them into operation at the commencement of an outbreak, preferring to wait and see if it becomes more serious. When the outbreak has become serious the time when the hospital would have been of most service has gone by.

The following are instances taken from published reports of medical inspectors of the Local Government Board after inspections made by them with reference to outbreaks of infectious disease or to the sanitary circumstances and administration of particular districts. Names are omitted, and it is only fair to say that in some of the districts other arrangements have since been made.

*Urban District; Population*, 5,000; *Date of Report*, 1907.—The hospital buildings are of corrugated iron and wood. They consist of a pavilion containing two wards and a duty room; a block containing mortuary, wash-house, and larder: and a block containing ambulance shed and disinfector shed. There is no administration block, and no caretaker resides on the premises. In consequence, when there are patients to be isolated, delay occurs in order to hire nurses, to air beds, etc., and the caretaker or nurse has to occupy one of the two wards. The wards contain four beds each. The mortuary has a large east window, and the larder is next door to it in the same block. Water is obtained from a deep well in the hospital grounds. Sewage is carried to a cesspit with overflow to an open ditch.

*Rural District; Population*, 13,000; 1906.—The so-called Infectious Diseases Hospital is a shed erected with planks and timber that had formed part of the huts built by a railway company to accommodate their navvies working in this locality. This shed has been placed in the corner of a meadow about four yards from a school. There is no road to it—indeed, there is not even any path but that which has been worn across the grass by persons going to and from the shed. There is no water supply, nor is there drainage of any kind, nor, indeed, any sanitary convenience save a roughly made portable one used with a pail. Refuse is thrown on the ground in the corner of a field. The walls of this shed are of rough boards, tarred; the roof is of boards covered with felt, over which is a sheet of corrugated iron; interiorly this roof is unceiled; the floor is of boards. The shed is divided by partitions of planks into three compartments, forming two wards placed on either side of a central compartment termed the nurses' room or kitchen. Nearly in the centre of this last-named compartment is a small central stove with chimney running straight up to the roof; this is for cooking and likewise for warming the whole building. Light is obtained from a small window in the front of each room; there is no back window or through ventilation of any kind. In this wooden building at night lamps or candles are used.

The whole is surrounded at a few feet distance by a low and climbable wooden fence. During the scarlet fever prevalence in 1905 one entire family and three mothers and children were removed to this building. The three last-mentioned families were accommodated together. No nurse or attendant was employed by the District Council to attend to the wants of these persons. The mothers of the sick children were taken into the hospital, and nursed their children and cooked for them and for themselves as they best could. Food was supplied them by local tradesmen under the doctor's supervision.

In the case of the first family admitted the whole family—viz., husband, wife, and four children—(one child suffering from scarlet fever) were transported to this building under a magistrate's order in respect of the child, the infected and contact cases being located together. After the family had been at the hospital a week another child fell ill of scarlatina, but the baby escaped infection altogether. The furniture and fittings were insufficient. Water was brought up about twice a week in a tank from the village. There was only one bath, but the inspector was unable to learn that much use was made of it for any purpose other than washing clothes. There were only two enamelled iron washing-bowls for the use of everyone. No sort of bathing was ordered before leaving the hospital.

(*To be continued.*)

